# Resveratrol as a Novel Therapeutic Approach for Diabetic Retinopathy: Molecular Mechanisms, Clinical Potential, and Future Challenges

**DOI:** 10.3390/molecules30153262

**Published:** 2025-08-04

**Authors:** Snježana Kaštelan, Suzana Konjevoda, Ana Sarić, Iris Urlić, Ivana Lovrić, Samir Čanović, Tomislav Matejić, Ana Šešelja Perišin

**Affiliations:** 1 Department of Ophthalmology, Clinical Hospital Dubrava, School of Medicine, University of Zagreb, 10000 Zagreb, Croatia; 2 Department of Ophthalmology, Zadar General Hospital, 23000 Zadar, Croatia; 3 Department of Health Studies, University of Zadar, 23000 Zadar, Croatia; 4 School of Medicine, Catholic University of Croatia, Ilica 242, 10000 Zagreb, Croatia; 5 Department of Ophthalmology, Clinical Hospital Dubrava, 10000 Zagreb, Croatia; 6 Health Center Zagreb East, 10000 Zagreb, Croatia; 7 Surgery Clinic, Clinical Hospital Sveti Duh, 10000 Zagreb, Croatia; 8 Department of Pharmacy, School of Medicine, University of Split, 21000 Split, Croatia

**Keywords:** diabetic retinopathy, resveratrol, oxidative stress, inflammation, angiogenesis, neuroprotection, mitochondrial dysfunction, autophagy, gut-retina axis, drug delivery systems

## Abstract

Diabetic retinopathy (DR) is a progressive, multifactorial complication of diabetes and one of the major global causes of visual impairment. Its pathogenesis involves chronic hyperglycaemia-induced oxidative stress, inflammation, mitochondrial dysfunction, neurodegeneration, and pathological angiogenesis, as well as emerging systemic contributors such as gut microbiota dysregulation. While current treatments, including anti-vascular endothelial growth factor (anti-VEGF) agents, corticosteroids, and laser photocoagulation, have shown clinical efficacy, they are largely limited to advanced stages of DR, require repeated invasive procedures, and do not adequately address early neurovascular and metabolic abnormalities. Resveratrol (RSV), a naturally occurring polyphenol, has emerged as a promising candidate due to its potent antioxidant, anti-inflammatory, neuroprotective, and anti-angiogenic properties. This review provides a comprehensive analysis of the molecular mechanisms by which RSV exerts protective effects in DR, including modulation of oxidative stress pathways, suppression of inflammatory cytokines, enhancement of mitochondrial function, promotion of autophagy, and inhibition of pathological neovascularisation. Despite its promising pharmacological profile, the clinical application of RSV is limited by poor aqueous solubility, rapid systemic metabolism, and low ocular bioavailability. Various routes of administration, including intravitreal injection, topical instillation, and oral and sublingual delivery, have been investigated to enhance its therapeutic potential. Recent advances in drug delivery systems, including nanoformulations, liposomal carriers, and sustained-release intravitreal implants, offer potential strategies to address these challenges. This review also explores RSV’s role in combination therapies, its potential as a disease-modifying agent in early-stage DR, and the relevance of personalised medicine approaches guided by metabolic and genetic factors. Overall, the review highlights the therapeutic potential and the key translational challenges in positioning RSV as a multi-targeted treatment strategy for DR.

## 1. Introduction

Diabetes mellitus and diabetic retinopathy (DR) are rapidly escalating global public health concerns. DR affects one in three individuals with diabetes and remains the leading cause of blindness among the adult working population, necessitating the provision of comprehensive eye care services for individuals living with diabetes [1,2]. According to the World Health Organization, the global number of people with diabetes is projected to be more than double, rising from 171 million in 2000 to 366 million by 2030, with a similar prevalence observed in both men and women, and the highest rates occurring in individuals aged 75–79 years. In 2021, global health expenditures related to diabetes were estimated at USD 966 billion, and are expected to increase to USD 1054 billion by 2045 [3,4]. Although the management of DR, a largely irreversible complication of diabetes mellitus, has become increasingly effective, it remains significantly more costly than the treatment of many other ocular diseases, placing a substantial burden on both patients and healthcare systems [2].

DR is a microvascular complication of diabetes mellitus that progresses gradually, typically presenting with worsening vision, the appearance of floaters, distorted visual perception, and, in advanced stages, partial or complete vision loss due to retinal detachment [5]. The severe forms of DR, which significantly affect vision, include proliferative DR, characterised by the abnormal growth of new retinal blood vessels, and diabetic macular oedema, marked by fluid accumulation and exudation in the central part of the retina [6]. The main risk factors for DR include chronic hyperglycaemia, hypertension, dyslipidaemia, obesity, duration of diabetes, and genetic predisposition, all of which contribute to the onset and progression of retinal microvascular damage [7,8,9,10,11]. In addition to managing and treating modifiable risk factors in patients with diabetes mellitus, the standard treatment for proliferative DR and/or diabetic macular oedema typically includes laser photocoagulation and/or intravitreal injections of anti-vascular endothelial growth factor (anti-VEGF) agents. These therapies often require multiple sessions to achieve and maintain optimal visual outcomes [12,13,14,15]. Intravitreal corticosteroid injections are commonly employed as second-line therapy. In more severe cases, complications such as persistent vitreous haemorrhage and tractional retinal detachment may necessitate a vitrectomy, a surgical procedure involving the removal of the vitreous humour and its replacement with a different solution [13].

Additionally, intravitreal aflibercept has received FDA approval for non-proliferative DR [14,15]. However, routine use of anti-VEGF therapy for non-proliferative DR remains unlikely as, despite initial regression of vascular lesions and temporary improvement in DR severity, studies indicate that underlying retinal ischemia remains unaltered and pathological changes often recur shortly after treatment cessation [16]. Anti-VEGF therapy is further limited by its short half-life and the need for frequent invasive intravitreal injections. While both photocoagulation and anti-VEGF agents have demonstrated efficacy in slowing disease progression, challenges such as drug resistance, suboptimal therapeutic responses, and the burdensome nature of repeated ocular injections continue to hinder treatment outcomes and patient adherence [17].

Given the current limitations of available DR therapies, primarily their invasive nature and limited efficacy in early disease stages, there is an urgent need to develop complementary or alternative therapeutic strategies [12]. These novel approaches should ideally target early pathogenic mechanisms such as oxidative stress, neuroinflammation, and mitochondrial dysfunction, and be delivered through non-invasive or less invasive routes to enhance patient compliance and therapeutic outcomes. Furthermore, interventions that improve the efficacy or durability of current treatments, while providing sustained protection against retinal neurovascular damage, are highly desirable. Accordingly, naturally derived bioactive compounds with pleiotropic properties have gained increasing attention. Among them, resveratrol (RSV), a polyphenol known for its antioxidant, anti-inflammatory, and neuroprotective effects, has emerged as a promising candidate for managing DR, either as an adjunct or a stand-alone therapy by modulating multiple pathological pathways involved in its progression [18,19,20].

**Figure 1:**
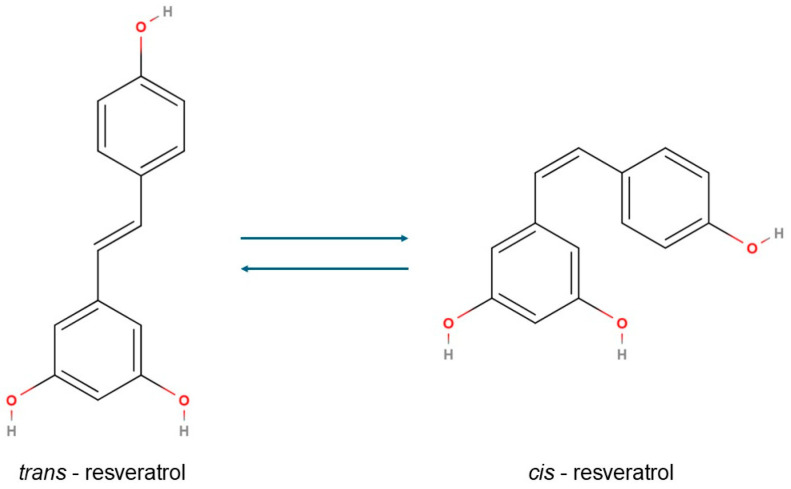
The chemical structure of the cis and trans isomers of resveratrol.

RSV (3,4′,5-trihydroxy-trans-stilbene), a naturally occurring polyphenol found in grapes, red wine, berries, peanuts, and certain medicinal plants, has gained significant attention for its diverse health-promoting effects, including anti-inflammatory, antioxidant, anticarcinogenic, and lipid-modulating properties (Figure 1). Due to its natural origin and chemical structure, resveratrol is classified as a natural polyphenolic compound belonging to the stilbene group. Structurally, it comprises two benzene rings: one bearing hydroxyl groups at the 3 and 5 positions, and the other with a hydroxyl group at the 4′ position. This specific substitution pattern defines resveratrol as a polyphenol, contributing to its strong antioxidant properties. The two aromatic rings are connected via a carbon–carbon double bond (C=C), which forms the characteristic stilbene backbone of the molecule. Resveratrol naturally occurs in two geometric isomers, trans and cis. Among these, the trans isomer is both thermodynamically more stable and biologically more active, whereas the *cis* isomer primarily arises from photoisomerisation or the thermal conversion of the trans form under exposure to light, heat, or alkaline pH [21,22,23].

RSV contributes to metabolic and vascular health by reducing vascular inflammation, enhancing vasodilation, inhibiting pathological angiogenesis, and improving glucose homeostasis. It also decreases insulin resistance, promotes autophagy, regulates lipid metabolism, protects pancreatic β-cells, alleviates diabetic cardiomyopathy, and upregulates glucose transporter type 4 (GLUT4) expression [24,25,26]. These pleiotropic actions are primarily mediated through activation of AMP-activated protein kinase (AMPK) and sirtuin 1 (SIRT1), which are key regulators of cellular energy homeostasis and stress resistance, thus supporting the prevention and management of diabetes-related complications [18]. In the retina, RSV has been shown to attenuate oxidative stress and prevent retinal ganglion cell apoptosis by activating the nuclear factor erythroid 2-related factor 2/heme oxygenase-1 (Nrf2/HO-1) signalling pathway [27]. As a master regulator of antioxidant defence, Nrf2 plays a central role in counteracting oxidative damage and maintaining glucose balance [28]. Despite these promising effects, the clinical translation of RSV has been hindered by its poor aqueous solubility, rapid metabolism, and low systemic bioavailability. Various nanotechnology-based drug delivery systems have been developed to overcome these challenges, including liposomes, polymeric nanoparticles, solid lipid nanoparticles, nanoemulsions, and exosome-based formulations. These strategies aim to improve RSV’s pharmacokinetic profile, increase retinal bioavailability, and enhance its therapeutic efficacy in vivo [29].

This review critically synthesises current evidence on the molecular mechanisms of RSV in DR, with an emphasis on its antioxidant, anti-inflammatory, and anti-angiogenic properties. It includes a discussion of preclinical findings, examines the results of emerging clinical studies, and analyses the potential of novel delivery systems to overcome existing pharmacological limitations. By synthesising recent advances, the aim is to assess the translational viability of RSV-based therapies in DR and outline prospective directions for future research and clinical application.

## 2. Pathophysiology of Diabetic Retinopathy

DR is a complex, multifactorial disease characterised by progressive neurovascular damage to the retina, driven by chronic hyperglycaemia, and exacerbated by oxidative stress, inflammation, neurodegeneration, and vascular dysfunction. While traditionally considered a microvascular complication of diabetes, recent advances have underscored the significant involvement of neurodegenerative processes, metabolic dysregulation, and gut-derived inflammatory mechanisms. Understanding these interconnected pathological pathways is essential for developing effective therapeutic strategies [30,31]. Table 1 summarises the key pathogenic mechanisms involved in DR, highlighting the principal molecular pathways, retinal impacts, and clinical relevance, along with the modulatory effects of RSV as a potential therapeutic agent [32,33,34,35,36,37,38,39,40,41,42,43,44,45,46,47,48,49,50,51,52,53,54].

One of the earliest pathogenic mechanisms in DR is the overproduction of reactive oxygen species (ROS) due to hyperglycaemia-induced mitochondrial dysfunction. High glucose levels disrupt the mitochondrial electron transport chain (ETC), leading to excessive ROS generation and mitochondrial DNA damage, including double-strand breaks (DSBs), which trigger the overactivation of poly(ADP-ribose) polymerase (PARP) [35]. Hyperactivated PARP depletes cellular NAD+ and poly(ADP-ribosyl)ates glyceraldehyde-3-phosphate dehydrogenase (GAPDH), thereby inhibiting glycolysis and rerouting glucose metabolism into alternative damaging pathways, such as the polyol pathway, protein kinase C (PKC) activation, and hexosamine biosynthesis, all of which contribute to the accumulation of advanced glycation end-products (AGEs) and amplify oxidative damage in retinal vascular and neural cells [36,37].

To counteract oxidative insults, the transcription factor Nrf2 plays a pivotal role. Under oxidative stress conditions, Nrf2 dissociates from its cytoplasmic inhibitor Keap1, translocates into the nucleus, and binds to antioxidant response elements (AREs), initiating the transcription of detoxifying and antioxidant genes such as HO-1, NQO1, and glutathione S-transferases [38]. This Nrf2/ARE signalling pathway constitutes a vital endogenous defence mechanism that attenuates ROS-induced tissue injury and preserves retinal homeostasis. However, chronic hyperglycaemia may impair Nrf2 activation over time, weakening the antioxidant defence system in the diabetic retina [39,40].

Parallel to oxidative stress, low-grade chronic inflammation plays an important role in the pathogenesis of DR. The nuclear factor kappa B (NF-κB) pathway, a central regulator of immune and inflammatory responses, is activated under prolonged hyperglycaemic conditions, leading to the upregulation of pro-inflammatory cytokines and chemokines [41]. Microglial cells, the resident immune cells of the retina, shift to an activated amoeboid phenotype and migrate from the inner to the outer retina, where they secrete inflammatory mediators including interleukin-1β (IL-1β), IL-6, tumour necrosis factor-alpha (TNF-α), and monocyte chemoattractant protein-1 (MCP-1) [42]. These molecules disrupt the integrity of the blood–retinal barrier (BRB), attract peripheral immune cells, and exacerbate local inflammation. Notably, gliosis, reactive changes in Müller cells, and astrocytes, are strongly associated with early neuronal loss and microvascular damage [43]. Müller cells, which encase all retinal vessels, regulate vascular permeability and retinal metabolism, and modulate glial responses and cell survival through cytokine release and neurotrophic support [44,45].

Neurodegeneration, an early and critical component of DR, is evidenced by the loss of retinal ganglion cells (RGCs), the thinning of the retinal nerve fibre layer (RNFL), and synaptic dysfunction. RGC apoptosis can precede the development of detectable microvascular lesions, indicating that neuronal damage may be the initiating factor in DR [46]. Moreover, excitotoxicity driven by excessive extracellular glutamate further contributes to neurodegeneration. Glutamate overstimulation activates N-methyl-D-aspartate receptors (NMDARs), leading to intracellular calcium overload, osmotic imbalance caused by Na^+^ and Cl^−^ influx, and ultimately neuronal swelling and death. In parallel, impaired autophagy, a cellular process essential for the clearance of damaged organelles and proteins, has been implicated in DR, further exacerbating oxidative damage and cell death [47,48,49].

Vascular abnormalities, including microaneurysms, capillary dropout, and neovascularisation, are hallmark features of DR. VEGF, a potent proangiogenic cytokine, is markedly upregulated in diabetic retinas and correlates with insulin resistance and elevated insulin-like growth factor-1 (IGF-1) levels [50,51]. VEGF functions with intercellular adhesion molecule-1 (ICAM-1) to promote leukocyte adhesion, endothelial dysfunction, and BRB breakdown. The expression of ICAM-1 is regulated through the PKC/endothelin (ET)/NF-κB axis, forming a signalling loop that perpetuates vascular inflammation and non-perfusion. Hypoxia-inducible factor-1 alpha (HIF-1α), stabilised under diabetic and hypoxic conditions, further amplifies VEGF expression and pathological angiogenesis, contributing to proliferative DR and vision-threatening complications [52,53,54].

Emerging research highlights the involvement of the gut–retina axis as a novel contributor to DR pathogenesis. Gut dysbiosis in diabetes disrupts intestinal barrier integrity, leading to metabolic endotoxemia and systemic inflammation. Circulating endotoxins, such as lipopolysaccharide (LPS), can cross the compromised BRB and activate retinal microglia and endothelial cells, further fuelling local oxidative and inflammatory responses. Altered gut microbiota may also affect host metabolism, short-chain fatty acid production, and immune modulation, indirectly impacting retinal health. Thus, gut-derived inflammatory signals and microbial metabolites could represent upstream modulators of DR pathophysiology, opening new avenues for therapeutic intervention-targeting systemic-metabolic crosstalk. Given its systemic influence, the gut–retina axis represents a promising therapeutic target. Modulating gut microbiota through prebiotics, probiotics, or dietary interventions may offer novel approaches to mitigate inflammation and improve retinal outcomes in DR [32,33,34].

## 3. Molecular Mechanisms of Resveratrol in Diabetic Retinopathy

RSV, a polyphenolic compound found in grapes, red wine, and various plant sources, exhibits significant therapeutic potential in addressing the complex pathophysiology of DR. Its effects span multiple molecular pathways, targeting oxidative stress, inflammation, neurodegeneration, angiogenesis, and metabolic dysfunction. By influencing key cellular signalling cascades, RSV offers a multifaceted approach to mitigating the progression of DR [18].

### 3.1. Antioxidant and Cytoprotective Effects

One of RSV’s central mechanisms in DR is its potent antioxidant activity. It exerts cytoprotective effects primarily through the activation of the Nrf2/ARE pathway. Activation of Nrf2 leads to the transcription of various antioxidant and phase II detoxification enzymes, which collectively diminish oxidative damage to cellular macromolecules, such as lipids, proteins, and DNA [55,56]. This contributes to reduced retinal oxidative stress, a key driver of DR pathogenesis.

Other naturally occurring Nrf2 activators, including sulforaphane, curcumin, allicin, epigallocatechin gallate (EGCG), quercetin, luteolin, and apigenin, have also been shown to offer cytoprotective effects by attenuating oxidative stress and inflammation [57,58,59,60]. However, despite their promising antioxidant potential, challenges related to low bioavailability and the risk of overactivating redox-sensitive transcription factors call for a cautious and regulated therapeutic approach [61].

Beyond the Nrf2/ARE pathway, RSV confers mitochondrial protection through activation of the AMPK/SIRT1/PGC-1α signalling axis. By stimulating AMPK and SIRT1, RSV enhances mitochondrial biogenesis and function, reduces reactive oxygen species (ROS) generation, and prevents apoptosis in retinal cells under hyperglycaemic conditions [62,63]. Moreover, AMPK activation contributes to improved insulin sensitivity and energy homeostasis, reinforcing its value in metabolic regulation [64,65]. The synergy between AMPK and SIRT1 underscores the comprehensive impact of RSV on mitochondrial health and metabolic control [66,67].

### 3.2. Anti-Inflammatory Mechanisms

Inflammation plays a pivotal role in the progression of DR, and RSV exerts strong anti-inflammatory actions by targeting key inflammatory mediators. One of the primary pathways influenced by RSV is the nuclear factor kappa-light-chain-enhancer of activated B cells (NF-κB) signalling pathway. By inhibiting NF-κB activation, RSV suppresses the expression of pro-inflammatory cytokines such as TNF-α, IL-1β, and IL-6, which are elevated in the diabetic retina and contribute to vascular damage and neural dysfunction [68].

Natural compounds, including bee venom, Vaccinium bracteatum extract, and licochalcone B, have also been reported to inhibit NF-κB activity, indicating that targeting this pathway may represent a viable anti-inflammatory strategy in DR [69,70,71].

RSV also modulates microglial activation, a central event in retinal neuroinflammation. Hyperactivation of microglia contributes to neuronal injury and degeneration in DR. Studies have shown that RSV and other agents, such as Nanog and Piper cubeba extract, suppress microglial activation by interfering with NF-κB signalling [72,73]. Moreover, RSV downregulates thioredoxin-interacting protein (TXNIP) and inhibits the NOD-like receptor pyrin 3 (NLRP3) inflammasome, a critical intracellular complex involved in the maturation of pro-inflammatory cytokines. This action reduces retinal vascular inflammation and protects against endothelial cell apoptosis [68].

### 3.3. Neuroprotection and Retinal Cell Survival

RSV contributes to the survival and protection of retinal neurons through several interconnected mechanisms. It significantly upregulates SIRT1, which helps mitigate oxidative and inflammatory insults. This upregulation supports the viability of retinal ganglion cells, as evidenced in both DR models and experimental optic neuritis [74,75,76].

Additionally, RSV promotes autophagy in Müller glial cells, which play a key role in maintaining retinal homeostasis. It achieves this through regulation of the miR-29b/SP1 signalling pathway, leading to increased expression of autophagy-related proteins such as LC3-I/II and Beclin-1 [77,78]. By enhancing autophagic flux, RSV reduces apoptotic cell death and preserves retinal structure and function. This autophagic activity is also modulated in part by SIRT1, indicating a synergistic effect between the two pathways in neuroprotection and cellular maintenance [79,80].

Furthermore, the activation of the Nrf2 pathway by RSV extends beyond antioxidant functions to confer direct neuroprotective and anti-ageing effects. Through this pathway, RSV shields retinal neurons from oxidative insults and supports long-term neural integrity [27].

### 3.4. Anti-Angiogenic Properties

A hallmark of advanced DR is pathological neovascularisation, driven primarily by upregulated VEGF and hypoxia-inducible factor 1-alpha (HIF-1α). RSV exerts anti-angiogenic effects by downregulating these key mediators, thereby impairing endothelial cell proliferation and migration, critical steps in aberrant angiogenesis [62].

Similar effects have been observed with bioactive agents such as nargenicin A1 and Persian shallot extract, which suppress VEGF signalling and inhibit pathological vascular responses [81,82]. Additionally, molecules such as death-associated protein kinase (DAPK) and suppressor of MEK1 (sMEK1) have been implicated in regulating HIF-1α expression, offering further insights into angiogenesis control mechanisms [70].

RSV also inhibits the endothelial-to-mesenchymal transition (EndMT), which contributes to vascular dysfunction in DR. This effect is achieved through inhibition of PKC-dependent NADPH oxidase activity, which limits ROS production and protects vascular cells from oxidative stress [83]. Furthermore, RSV modulates nitric oxide synthase (NOS) activity, supporting vascular tone and endothelial health. Nevertheless, some uncertainty persists regarding RSV’s dose-dependent effects on angiogenesis, with conflicting results in preclinical studies highlighting the need for standardised dosing protocols [84].

### 3.5. Broader Relevance in Retinal Vascular Pathology

Although primarily investigated in the context of DR, RSV also exhibits protective effects in other retinal vascular conditions. For instance, in models of retinal ischemia/reperfusion injury, RSV modulates the expression of matrix metalloproteinase-9 (MMP-9), inducible nitric oxide synthase (iNOS), and heme oxygenase-1 (HO-1), contributing to reduced vascular leakage and cell death [85]. These findings underscore the broader therapeutic applicability of RSV to various retinal diseases characterised by oxidative stress and vascular compromise.

RSV represents a promising multi-targeted therapeutic agent for DR. Through modulation of interconnected molecular pathways, such as Nrf2/ARE, AMPK/SIRT1, NF-κB, and VEGF/HIF-1α, RSV addresses the multifaceted nature of DR, influencing oxidative damage, inflammation, neurodegeneration, angiogenesis, and metabolic imbalances [79,80]. Despite encouraging preclinical outcomes, the translation of RSV into clinical practice demands further exploration of its pharmacokinetics, dosage optimisation, bioavailability, and long-term safety profiles.

### 3.6. Structural Activity Relationship (SAR) of Resveratrol: Structural Domains and Functional Implications

The biological activity of RSV in diabetic retinopathy is closely linked to specific structural features within its molecule. In the context of SAR analysis, the molecular framework of RSV can be conceptually divided into three key structural domains: the planar aromatic rings, the hydroxyl (phenolic) functional groups, and the central ethylene bridge. The 3,5,4-trihydroxy substitution pattern is central to its antioxidant potential, enabling efficient scavenging of ROS, which are typically elevated in diabetic retinal tissue. Substitution or removal of these hydroxyl groups significantly reduces RSV’s biological efficacy. Among these, the 4-hydroxyl group is particularly crucial for inhibiting pro-inflammatory cytokines such as IL-1β and TNF-α through modulation of the NF-κB signalling pathway [23,86]. Structural derivatives containing additional hydroxyl groups, such as piceatannol, have demonstrated enhanced inhibitory effects on COX-2 and NF-κB, both key mediators in the pathogenesis of diabetic retinopathy [87,88]. The planar aromatic rings are essential for π-π interactions with various biomolecular targets, including enzymes and membrane proteins involved in inflammation and oxidative stress pathways. The planar conjugated structure of RSV facilitates membrane permeability and allows it to modulate intracellular signalling cascades such as NF-κB, Nrf2/ARE, and VEGF/PI3K-Akt pathways—key regulators in the pathogenesis of diabetic retinopathy. The ethylene bridge, with its trans configuration, imparts structural rigidity and electronic delocalisation, which are crucial for maintaining bioactivity [23]. Furthermore, structural modifications such as methoxylation (e.g., pterostilbene) have been shown to improve lipophilicity and bioavailability, enhancing the compound’s apoptotic and antioxidant effects in retinal cells. Additionally, glycosylated derivatives like piceid (resveratrol-3-O-glucoside) offer increased in vivo stability and more effective systemic delivery, providing additional protection to retinal tissue [86,89,90].

Collectively, these structural features are responsible for RSV’s broad spectrum of biological activities, including anti-inflammatory, antioxidant, neuroprotective, and antiangiogenic effects, all of which are relevant in the pathophysiology of diabetic retinopathy.

## 4. Challenges in the Clinical Translation of Resveratrol

RSV, a naturally occurring polyphenol with demonstrated antioxidant, anti-inflammatory, and anti-angiogenic properties, has shown compelling preclinical efficacy in the treatment of retinal diseases such as DR. However, despite extensive preclinical evidence supporting its therapeutic potential, the clinical translation of RSV remains significantly limited. These limitations stem primarily from pharmacokinetic challenges, variability in treatment response, and a lack of robust clinical evidence, all of which hinder its integration into routine ocular therapeutics.

### 4.1. Poor Bioavailability and Rapid Metabolism

A fundamental challenge in the clinical application of RSV is its inherently poor bioavailability. RSV is classified as a BCS Class II compound, characterised by low aqueous solubility and high membrane permeability, which necessitates formulation strategies to enhance its bioavailability [91]. It has high oral absorption due to its lipophilicity; however, due to rapid and extensive metabolism in the liver and intestines, only small amounts of unchanged RSV reach systemic circulation. In the bloodstream, it appears in free form or as glucuronide and sulphate conjugates, with the free form primarily binding to serum albumin, which serves as a potential reservoir for RSV capable of binding it and thereby influencing its distribution and bioavailability [90]. The stability of the RSV–albumin complex and its cellular uptake via albumin and LDL receptors further confirms its absorption and transport mechanisms in the body [22,92]. This results in subtherapeutic concentrations at the target site, particularly in ocular tissues that are protected by the blood–retinal barrier. The inability of RSV to efficiently penetrate this barrier further reduces its therapeutic efficacy in the posterior segment of the eye [92].

These pharmacokinetic limitations are not unique to RVS. Compounds such as Akebia saponin D (ASD) also exhibit ultra-low bioavailability due to poor gastrointestinal permeability and extensive pre-absorption degradation [93]. Similarly, drugs like ketamine and clarithromycin are subject to significant hepatic metabolism [94,95]. In contrast, structural modification strategies have been employed to enhance the bioavailability of other therapeutic agents, such as chaplain inhibitors, which have demonstrated improved retinal penetration and sustained drug levels [96].

### 4.2. Innovations to Enhance Bioavailability

To overcome these limitations, various drug delivery innovations have been explored. Nanoencapsulation, chemical derivatisation, including hydroxylation and glycosylation, and lipid-based formulations have demonstrated improved solubility, enhanced systemic distribution, and increased metabolic stability of RSV [22,92]. Micronisation and nanotechnology-based delivery systems, such as liposomes and polymeric nanoparticles, have further enhanced its pharmacokinetic profile by improving tissue penetration and prolonging intraocular retention [97].

Additional strategies aimed at improving bioavailability include enhancing lipid solubility and intestinal permeability, as well as modulating gut flora to minimise presystemic metabolism [93,98]. However, the success of these strategies is highly dependent on the physicochemical and metabolic properties of the compound in question, and their applicability must be evaluated on a case-by-case basis.

### 4.3. Precision Dosing and Pharmacokinetic Variability

Effective therapy for retinal diseases such as DR necessitates precise dosing strategies due to the pharmacokinetic variability seen across patient populations. The presence of biological barriers, including the blood–retinal barrier and active efflux transporters, pose significant challenges to drug delivery in the posterior segment of the eye [59,99,100]. Moreover, interindividual differences in metabolism and systemic absorption can significantly alter therapeutic outcomes [99], underscoring the need for personalised dosing regimens. Personalised approaches that incorporate genetic, phenotypic, and metabolic profiles are essential to optimise treatment efficacy while minimising adverse effects [99,101].

### 4.4. Limitations in Clinical Evidence

Despite encouraging findings from preclinical models, the clinical application of RSV in retinal diseases remains largely theoretical due to the absence of large-scale, well-controlled clinical trials. Studies in animal models of age-related macular degeneration (AMD) and DR have shown antioxidant, anti-inflammatory, and anti-angiogenic effects [62,78,102], yet these results are not consistently replicated in human trials. Of the 165 clinical studies investigating RSV, the majority have focused on systemic metabolic conditions, with relatively few addressing ocular applications [103].

This gap in evidence is exacerbated by what has been termed the “Resveratrol Paradox”, a disconnect between its strong in vitro and animal model efficacy and its limited systemic availability and inconsistent performance in humans [104]. Furthermore, many preclinical studies utilise concentrations of RSV that are not physiologically achievable in humans, thereby limiting their translatability. The diversity in study methodologies and the use of proprietary formulations further complicate comparisons between trials and delay the development of standardised treatment protocols [105].

### 4.5. Safety Considerations and Systemic Implications

Although RSV is generally considered safe and well-tolerated, there is growing concern about its long-term safety and potential interactions with medications commonly used in DR patients, particularly those with diabetes. RSV may interact with antidiabetic drugs, potentially altering glycaemic control or affecting systemic metabolism and cardiovascular function [106]. Moreover, sudden improvements in glycaemic control, whether drug-induced or diet-mediated, have been linked to transient worsening of DR in some patients, further emphasising the need for caution when integrating RSV into treatment regimens.

Broader implications for DR management also need to be considered. Systemic therapies such as ACE inhibitors and angiotensin receptor blockers have demonstrated protective effects against DR progression [107], while anti-VEGF agents remain the cornerstone for managing neovascular complications [108,109]. Given the role of chronic inflammation and metabolic dysfunction in DR pathogenesis, systemic agents such as hypoglycaemics and lipid-lowering medications may provide ancillary benefits in reducing disease burden [106,110]. Nevertheless, any long-term strategy incorporating RSV must be rigorously tested in well-designed trials to confirm its safety and efficacy.

Although RSV holds substantial therapeutic promise, particularly due to its pleiotropic pharmacological effects, its clinical utility in retinal diseases remains limited by challenges related to poor bioavailability, rapid metabolism, dosing variability, and insufficient human data. Ongoing advances in drug delivery platforms, combined with personalised medicine approaches, offer avenues to overcome these barriers. However, the successful clinical translation of RSV will ultimately depend on the development of standardised formulations and the implementation of large-scale, randomised clinical trials that can validate its therapeutic potential in real-world patient populations.

## 5. Advances in Resveratrol Drug Delivery for Ocular Use

RSV’s therapeutic application in ocular diseases is limited by its pharmacokinetic properties and the anatomical barriers of the eye. Advances in drug delivery technologies aim to overcome these challenges by improving bioavailability, stability, and targeted release within ocular tissues [24,82,83,96,97,98].

### 5.1. Resveratrol Delivery Strategies and Routes of Administration in Ocular Use

Despite the promising effects of RSV in managing retinal dysfunction, its clinical translation has been significantly hindered by intrinsic pharmacokinetic limitations, including poor aqueous solubility, rapid systemic metabolism, and low oral bioavailability. These factors severely limit its therapeutic potential, especially in chronic conditions such as DR, where sustained exposure of the retina to active compounds is essential [24].

To overcome these limitations, various nanotechnology-based drug delivery systems have been developed. These include liposomes, polymeric nanoparticles, solid lipid nanoparticles, nanoemulsions, and exosome-based formulations. Such systems are designed to enhance RSV’s pharmacokinetic profile, increase its bioavailability in retinal tissues, and ultimately improve its therapeutic efficacy in vivo [24,82,83]. These advanced formulations also provide protection against premature degradation and offer controlled and targeted release features particularly advantageous in ocular drug delivery.

In addition to formulation strategies, multiple routes of administration have been investigated to optimise the delivery of RSV to retinal tissues. These include systemic, localised, and transmucosal approaches, each offering distinct benefits and presenting specific limitations.

Intravitreal injection delivers RSV directly into the vitreous cavity, achieving high local drug concentrations. Preclinical studies have demonstrated that intravitreal RSV administration significantly reduces vascular distortion and pathological neovascularisation in models of DR [38]. However, due to its invasive nature and potential for injection-related complications, this route may not be feasible for long-term or routine clinical use.

Topical instillation in the form of eye drops offers a non-invasive alternative. It has shown comparable benefits to intravitreal injection in diabetic models, including reduced retinal inflammation and preservation of retinal structure [38]. Nonetheless, effective transcorneal delivery remains a challenge, with limited retinal penetration and the need for frequent administration to maintain therapeutic levels.

Oral administration is the most patient-compliant route; however, it is associated with significantly reduced bioavailability due to extensive first-pass hepatic metabolism and poor intestinal absorption. Despite these limitations, long-term oral administration of RSV (5–10 mg/kg/day) has led to improvements in retinal function and vascular integrity in diabetic rats [107], suggesting that systemic delivery can still yield therapeutic benefits under optimised dosing regimens.

Sublingual delivery has emerged as a promising route to bypass hepatic first-pass metabolism and enhance systemic absorption of RSV. Novel formulations, such as fast-disintegrating sublingual mini-tablets, have been developed to improve bioavailability and enhance patient adherence [107]. Although still in the early stages of clinical development, this route offers a practical and less invasive alternative to oral and intravitreal administration.

Taken together, these alternative delivery strategies aim not only to overcome the pharmacokinetic challenges of RSV but also offer opportunities to tailor therapeutic approaches based on disease severity, patient-specific factors, and treatment goals. Importantly, by enhancing local or systemic bioavailability, RSV-based therapies have the potential to complement existing treatment modalities for DR, including anti-VEGF agents and corticosteroids, particularly in cases where neurovascular protection and long-term disease modulation are required.

### 5.2. Challenges of Topical Delivery and Novel Resveratrol-Based Ophthalmic Formulations

Topical ocular drug delivery, primarily through eye drops or ointments, accounts for approximately 90% of the global ophthalmic drug market and is the most widely used method for administering ophthalmic drugs due to its simplicity, non-invasiveness, and high patient compliance [111,112]. However, it has some limitations. The main disadvantage of conventional eye drops is the administration of an excessive fluid volume (20–70 µL), which exceeds the physiological capacity of the conjunctival sac (7–10 µL) [111,112]. This leads to overflow, reflex blinking, and increased drainage through the nasolacrimal duct. Additionally, rapid tear turnover significantly reduces the duration of drug contact with the ocular surface to 14–17 min, with most of the drug eliminated within the first 5 min [111,112].

Given that the drug concentration on the ocular surface decreases by approximately 16% per minute after administration, the formulation becomes diluted by a factor of 1:1000 within 40 min [111,112]. As a result, no more than 5% of the topically applied drug reaches the anterior ocular tissues, and usually less than 1% of the administered dose reaches the retina and vitreous [113]. This poor penetration is due to multiple physiological barriers, such as the tear film, blinking, nasolacrimal drainage, and the blood–retinal barrier. Therefore, posterior segment diseases such as DR are commonly treated with intravitreal injections or advanced delivery systems (e.g., nanoparticles, implants) that ensure prolonged and targeted drug release [111,112].

In response to these limitations, recent innovations in ocular drug delivery technologies have focused on overcoming low drug bioavailability, particularly for compounds like RSV, which exhibit poor solubility and rapid metabolism. By improving solubility, chemical stability, tissue permeability, and sustained release within ocular structures, these strategies enhance RSV’s therapeutic potential and clinical relevance in treating eye diseases such as DR. An overview of innovative topical formulations with RSV developed for ocular administration in the management of DR and other ocular diseases is presented in Table 2 [114,115,116,117,118,119,120,121,122,123,124,125,126,127].

### 5.3. Nanoformulations for Improved Bioavailability

A significant area of progress lies in the development of nanoformulations, including liposomes, nanoemulsions, polymeric nanoparticles, and self-nanoemulsifying drug delivery systems (SNEDDS). These nanocarriers have demonstrated considerable promise in enhancing the pharmacokinetic profile of RSV.

Liposomes and nanoemulsions have been shown to significantly improve the permeability of RSV across ocular barriers, thereby increasing its bioavailability [29]. Polymeric nanoparticles offer the additional advantage of enabling controlled drug release and prolonged retention within ocular tissues, contributing to more effective and sustained therapeutic outcomes [121]. SNEDDSs, on the other hand, provide exceptional cytocompatibility and a capacity for prolonged release while protecting RSV from enzymatic degradation [125]. These systems support targeted ocular drug delivery, minimise systemic side effects, and significantly enhance drug stability [123]. However, despite these benefits, concerns about potential toxicity, the need for extensive in vivo validation, and regulatory hurdles remain pressing challenges for clinical translation.

### 5.4. Sustained-Release Intravitreal Implants

Another promising advancement in ocular drug delivery is the use of sustained-release intravitreal implants, particularly for managing chronic conditions like DR. These delivery systems, including microspheres and hydrogels, are designed to reduce the frequency of invasive intravitreal injections by providing extended and controlled drug release.

Microspheres are biodegradable carriers that encapsulate therapeutic agents and release them gradually over time. This allows for precise control of drug kinetics, enhanced entrapment efficiency, and improved therapeutic consistency. Clinical applications of microspheres include the delivery of anti-VEGF agents and corticosteroids such as triamcinolone acetonide, which have been shown to reduce inflammation and inhibit neovascularisation in DR patients [124,125].

Temperature-sensitive hydrogels also offer a viable approach for sustained intraocular drug delivery. These hydrogels exhibit high biocompatibility and, upon injection, form semi-solid structures within the vitreous cavity. These delivery systems provide sustained drug release over prolonged periods while maintaining compatibility with the intraocular environment [126,127]. Together, microspheres and hydrogels can significantly reduce the frequency of intravitreal injections, improving patient adherence and lowering the risk of complications associated with repeated ocular procedures [128]. Nevertheless, long-term safety, targeting efficiency, and the challenge of penetrating the blood–retinal barrier remain areas that require further research and technological refinement.

### 5.5. Combination Therapies with Existing DR Treatments

The use of combination therapies in the treatment of DR is gaining traction to enhance therapeutic outcomes by leveraging the synergistic effects of multiple interventions. One such example is the combination of intravitreal triamcinolone with phacoemulsification, which has demonstrated significant improvements in visual acuity and a reduction in macular thickness, with no increase in adverse events [129]. Another promising combination is panretinal photocoagulation (PRP) with anti-VEGF therapy. This strategy has demonstrated superior outcomes in terms of neovascular regression and improvement in visual function compared to PRP alone, with no additional complications reported [130,131].

Although recent findings are promising, variability in study design, patient populations, and follow-up durations complicates direct comparisons across studies [12,13,16,17,18,132,133,134,135,136,137,138,139,140,141,142]. Moreover, while combination therapies hold potential to reduce the frequency of anti-VEGF injections, definitive evidence supporting their efficacy is still lacking [133]. Therefore, large-scale, standardised clinical trials are essential to confirm these results and facilitate their integration into routine clinical practice. Current standard therapies for DR are primarily directed at advanced disease stages and often fail to adequately address early pathophysiological processes such as oxidative stress, inflammation, and neurodegeneration. RSV, with its broad spectrum of biological activity, has emerged as a promising adjunct by targeting multiple molecular pathways implicated in DR. Table 3 presents a comparative overview of the mechanisms, benefits, and limitations of existing DR therapies, along with the potential complementary or enhancing role of RSV [12,13,14,15,16,50,51,52,53,54,55,56,57,58,59,60,61,62,63,64,65,66,67,68,69,70,71,72,73,74,75,76,77,78,79,80,129,130,131,132,133].

### 5.6. Need for Well-Designed Randomised Clinical Trials

To establish optimal dosage and treatment regimens for RSV-based and combination therapies, there is a critical need for well-designed randomised clinical trials (RCTs). These trials must address several key parameters, including controlling for variability in patient subgroups, follow-up durations, and study methodologies. Additionally, RCTs should rigorously evaluate the synergistic potential of combination treatments and assess their impact on injection frequency and long-term safety.

Confirmation of therapeutic efficacy through such trials will be instrumental in transitioning current experimental successes into validated, evidence-based treatment protocols for DR.

### 5.7. Personalised Medicine Approaches

An emerging frontier in the treatment of DR involves personalised medicine approaches, which tailor interventions based on individual genetic, phenotypic, and clinical profiles. This strategy promises more targeted and effective treatment regimens by accounting for variability among patients.

Genetic research has identified specific variants associated with DR susceptibility, particularly those involved in angiogenesis and inflammatory pathways [134]. By identifying these markers early, clinicians can initiate preventive or therapeutic interventions tailored to individual risk profiles.

Moreover, the DR management increasingly involves a multifactorial approach, with systemic control of glucose, blood pressure, and lipid levels being essential for overall disease management [13,15,135]. Personalised treatment strategies account for comorbidities and genetic predispositions, thereby improving outcomes and reducing the risk of progression [142].

Emerging therapeutics are now targeting specific disease pathways and components of the retinal neurovascular unit, including VEGF inhibitors and agents that modulate interactions between neurons, glia, and vasculature [141,143]. Machine learning tools have further enabled the identification of biomarkers, such as HDL/LDL levels, that predict differential responses to intensive glycaemic control, providing a basis for extending similar approaches to DR therapies [144].

Notably, individuals with type 2 diabetes who exhibit specific metabolic traits may respond more favourably to RVS-based interventions, suggesting the importance of personalised selection criteria [135,145]. Despite these advancements, challenges remain in implementing personalised approaches at scale, including the integration of multi-omic data, high costs, and limited accessibility across diverse patient populations.

### 5.8. Exploring Synthetic Analogues and Prodrugs of Resveratrol

To address the intrinsic limitations of RSV, namely its low solubility, instability, and rapid metabolism, researchers have been exploring the development of synthetic analogues and prodrug formulations. These strategies aim to retain or enhance the biological activity of RSV while improving its stability and pharmacological properties.

Synthetic analogues are structurally modified compounds designed to replicate the therapeutic effects of RSV but with enhanced bioavailability and chemical stability [139]. Prodrugs, on the other hand, involve the chemical modification of RSV. Prodrugs are inactive or less active compounds that convert into active forms within the body. For RSV, prodrug strategies aim to overcome its poor oral bioavailability, low stability, and rapid metabolism. Key goals include protecting phenolic hydroxyl groups from oxidation, enhancing chemical stability, and enabling targeted delivery to tissues such as the brain, retina, or liver. Various resveratrol prodrugs have been developed and synthesised from RSV: ester derivatives (e.g., acetate, butyrate) improve membrane permeability, glycosylated forms like piceid increase solubility and act as reversible carriers, phosphate prodrugs enhance water solubility, lipid conjugates facilitate CNS delivery, and nanoparticulate systems improve stability and targeting. These approaches aim to improve systemic and ocular delivery by enhancing the compound’s resistance to metabolic degradation and potentially advance the therapeutic potential of resveratrol in clinical use [146,147,148,149]. Recent studies have investigated RSV prodrugs with improved retinal delivery in diabetic retinopathy. One example is piceid-octanoate (PIC-OCT), a lipophilic ester of piceid designed to enhance bioavailability and target retinal tissue. In vitro and in vivo models have shown that PIC-OCT reduces oxidative stress, protects photoreceptors, and modulates SIRT1 and PARP1 pathways, highlighting its potential as a retina-targeted therapeutic [135].

**Figure 2:**
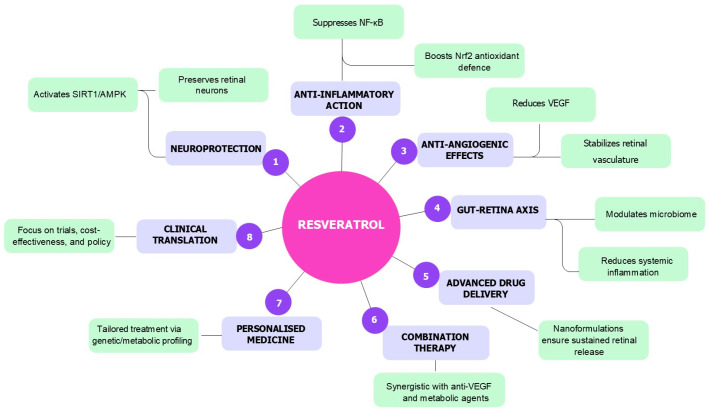
Overview of the multifactorial therapeutic actions of RSV in DR: RSV exerts neuroprotective, anti-inflammatory, and anti-angiogenic effects through activation of SIRT1/AMPK and Nrf2 pathways, suppression of NF-κB and VEGF signalling, and modulation of the gut–retina axis. Advanced delivery systems, combination therapies, and personalised medicine approaches further support its translational potential. Clinical translation requires well-designed trials and policy integration.

These developments represent a crucial step toward translating the promising outcomes observed in preclinical studies into clinically effective therapies. When used in conjunction with advanced drug delivery systems, such as nanoformulations and sustained-release platforms, synthetic analogues and prodrugs may significantly improve the clinical applicability of RSV for ocular diseases. To provide a synthesis of the pleiotropic therapeutic actions and translational strategies involving RSV in DR, Figure 2 summarises its multifaceted effects, including molecular mechanisms, delivery innovations, combinatory strategies, and directions toward personalised and clinical application [16,22,27,28,29,57,64,65,66,69,70,71,72,73,74,75,76,77,78,79,104,105,106,107,108,109,110,124,125,126,127,128,134,135,136,137,138].

## 6. Future Directions and Clinical Perspectives

Despite strong preclinical evidence supporting RSV as a multi-targeted compound with antioxidative, anti-inflammatory, neuroprotective, and anti-angiogenic properties, its clinical translation for DR remains limited. To bridge this gap, future research must focus on expanding insights, overcoming bioavailability barriers, validating therapeutic efficacy through clinical trials, and integrating RSV into personalised and combinatory treatment strategies [18,137].

Further elucidation of RSV’s molecular mechanisms is essential to unlock its full therapeutic potential in DR. While key signalling pathways, such as AMPK/SIRT1, NF-κB, Nrf2, and VEGF, are established mediators of RSV’s protective effects, additional regulatory layers remain underexplored [27,28,67,68]. Integrative multi-omic approaches, encompassing transcriptomics, proteomics, epigenomics, and metabolomics, should be employed to identify novel molecular signatures predictive of therapeutic response. Particular focus should be placed on mitochondrial biogenesis regulators, non-coding RNAs, and inflammation-resolution mediators, which may serve as next-generation targets for RSV-guided interventions in DR [20,138].

Emerging evidence positions DR as a neurovascular disease, where neurodegeneration often precedes and exacerbates microvascular dysfunction. RSV’s ability to protect retinal neurons, particularly ganglion cells, by modulating oxidative stress, enhancing autophagy, activating SIRT1, and suppressing microglial-mediated inflammation, supports its application as a neuroprotective agent [27,139,140]. Future preclinical and clinical studies should evaluate its capacity to preserve retinal function using electrophysiological metrics such as ERG, measurements of the OCT-based ganglion cell complex, and markers of retinal neuroinflammation. These endpoints may redefine RSV as a disease-modifying agent rather than merely symptomatic relief.

One of the primary limitations hindering the clinical applicability of RSV is its poor bioavailability caused by extensive hepatic metabolism and limited aqueous solubility [92,150]. To address this, several innovative drug delivery platforms are being developed, including liposomes, micelles, polymeric nanoparticles, nanoemulsions, and SNEDDS. Intravitreal sustained-release systems such as microspheres and hydrogels, as well as ROS/glucose-sensitive carriers, represent next-generation solutions for site-specific, long-acting retinal delivery [22,151,152,153]. These approaches must now be tested in long-term animal models and early-phase clinical studies to assess pharmacodynamics, retinal tissue penetration, and safety profiles under chronic use scenarios.

Another strategic avenue lies in integrating RSV into existing treatment regimens. Anti-VEGF agents, although effective, require frequent intravitreal injections and fail to address the oxidative and neuroinflammatory components of DR. RSV, by reducing VEGF expression and restoring BRB integrity, may reduce the frequency of injections and enhance long-term outcomes [154,155,156].

Moreover, the combination of RSV with neuroprotective agents, including citicoline, brimonidine, or metabolic modulators such as metformin and GLP-1 receptor agonists, may offer additive or synergistic effects. RSV could also mitigate corticosteroid-induced fibrosis by exerting anti-fibrotic effects [18,157]. These multidimensional therapeutic strategies represent a compelling frontier for clinical research. Future studies should explore the sequencing, dosing, and pharmacodynamic interactions of RSV with standard and emerging DR therapies to set future guideline-based implementation. To synthesise the clinical and molecular mechanisms underlying RSV’s therapeutic role in DR, Table 4 provides a structured overview of major pathophysiological pathways, molecular targets, proposed mechanisms of action, strategic therapeutic approaches, and their potential clinical implications [18,22,27,32,33,34,42,43,50,51,52,53,54,79,80,84,91,92,93,94,95,96,135,150,154,155,156,158].

Recent studies have linked diabetic gut dysbiosis with retinal pathology via the gut–retina axis. This bidirectional communication is driven by increased intestinal permeability, LPS-induced systemic inflammation, and altered production of short-chain fatty acids (SCFAs), all of which contribute to retinal endothelial dysfunction. RSV has demonstrated prebiotic properties, restoring microbial diversity, reducing metabolic endotoxemia, and attenuating systemic oxidative stress [33,34,159]. This systemic effect may indirectly protect the retina, opening new avenues for dual-targeted interventions that combine RSV with microbiota-directed therapies, including prebiotics, probiotics, and synbiotics [33,34,159,160]. Investigating gut-derived biomarkers such as SCFA profiles and endotoxin levels concerning RSV efficacy may provide a holistic framework for modulating retinal disease.

To date, the majority of RSV research in DR has been confined to preclinical models [135,150,161,162]. Existing clinical studies suffer from small sample sizes, heterogeneity in formulations, and a lack of standardised endpoints [18]. Future randomised controlled trials must stratify participants by DR stage, systemic comorbidities, and molecular biomarkers to assess efficacy more precisely.

Importantly, interindividual variability in RSV metabolism, systemic inflammation, and genetic background underscores the need for a precision medicine approach. Integrating metabolomic and genomic profiling with AI-assisted patient stratification may identify “RSV responders” and facilitate individualised therapeutic regimens. Additionally, simulation models based on systems biology could predict disease progression and optimise RSV-based intervention timing.

Although classified as a nutraceutical, RSV occupies a unique position at the interface between dietary supplements and pharmaceutical agents. Standardisation of formulation, quality control, and therapeutic dosing remains imperative before regulatory endorsement and inclusion in clinical guidelines. Incorporating RSV into eHealth and mHealth platforms for diabetes care may enhance treatment adherence, improve patient education, and enable remote monitoring of visual and systemic parameters [163,164]. Pharmacoeconomic studies should also evaluate the cost-effectiveness of RSV-based interventions in reducing DR-related morbidity and healthcare burden.

Given its favourable safety profile, RSV may be especially suitable for early-stage DR or high-risk diabetic populations with subclinical retinal changes. However, longitudinal data are needed to confirm its long-term impact on visual acuity, retinal integrity, and systemic metabolism.

## 7. Conclusions

DR remains a leading cause of vision impairment worldwide, driven by a complex interaction of metabolic, inflammatory, vascular, and neurodegenerative processes [1,2,3,4,25,26]. RSV, a bioactive polyphenol with multiple actions, stands out as a promising therapeutic candidate able to target several pathogenic pathways. RSV’s capacity to attenuate oxidative stress, modulate chronic inflammation, preserve neuronal integrity, regulate angiogenic signalling, and potentially influence gut–retina axis communication suggests that its effects extend beyond those of a conventional antioxidant [19,24,27,28,29]. These multifaceted actions position it as a systemically active, disease-modifying therapeutic agent. However, unlocking this potential requires overcoming key translational hurdles. Advanced drug delivery systems must address pharmacokinetic issues, while comprehensive clinical trials are needed to determine optimal dosing and long-term efficacy. Incorporating omics-based diagnostics and personalised medicine strategies will further improve patient selection and therapeutic predictability. Future research should also explore how RSV works synergistically with existing DR treatments, aiming to lessen treatment burden, improve functional outcomes, and probably delay disease progression.

With technological and scientific advances at hand, it is the right time to move RSV from promising preclinical findings to a validated part of integrated DR therapy. Supported by strong mechanistic understanding and emerging translational innovations, RSV could ultimately change how we prevent and treat the neurovascular complications of diabetes, potentially safeguarding vision and establishing a new approach to metabolic and retinal health.

## Figures and Tables

**Table 1:** Pathogenic Mechanisms and Retinal Changes in Diabetic Retinopathy: Resveratrol’s Therapeutic Relevance.

Pathogenic Mechanism	Key Molecular Players	Pathophysiological Impact on Retina	Clinical Relevance	Therapeutic Effects of Resveratrol
Oxidative stress and mitochondrial dysfunction	ROS, ETC, PARP, NAD^+^, GAPDH, Nrf2, ARE	-Hyperglycaemia-induced ROS damages mitochondria and DNA, overactivates PARP, impairs NAD^+^/energy metabolism, and suppresses Nrf2-mediated antioxidant responses, leading to endothelial/pericyte dysfunction	-Precedes clinical DR signs -Correlates with early microvascular damage -Therapeutic window for antioxidants.	-Activates Nrf2/ARE pathway, enhances HO-1, NQO1 expression, and stimulates AMPK/SIRT1/PGC-1α signalling to reduce ROS, -Preserve mitochondrial function -Inhibit apoptosis.
Chronic inflammation and cytokine activation	NF-κB, TNF-α, IL-1β, IL-6, MCP-1, ICAM-1	NF-κB activation increases inflammatory cytokines, microglial activation, BRB breakdown, and leukostasis; causes oedema and capillary damage	-Drives NPDR and PDR associated with retinal thickening and macular oedema -Rationale for corticosteroids and anti-inflammatory therapy	-Inhibits NF-κB signalling -Downregulates TNF-α, IL-1β, IL-6, and MCP-1 -Suppresses microglial activation -Blocks NLRP3 inflammasome via TXNIP inhibition
Neurodegeneration and apoptosis	RGCs, Müller cells, glutamate, NMDAR, Ca^2+^, autophagy proteins	RGC apoptosis, RNFL thinning, excitotoxicity via glutamate/NMDAR → Ca^2+^ influx -Impaired autophagy leads to neuroinflammation and cell loss	-Occurs early, even before vascular damage -Causes visual dysfunction -Supports neuroprotective strategies	-Upregulates SIRT1 and promotes autophagy (LC3-II, Beclin-1) -Activates Nrf2 for neuroprotection, -Maintains Müller cell function
Pathological angiogenesis	VEGF, IGF-1, PKC, ICAM-1, HIF-1α	-Hypoxia and hyperglycaemia upregulate VEGF/HIF-1α, leading to neovascularisation and BRB breakdown -ICAM-1 promotes leukocyte adhesion.	-Hallmark of PDR and DME -Treated with anti-VEGF agents -Signs include haemorrhages, exudates, neovascularisation	-Downregulates VEGF and HIF-1α -Inhibits EndMT via PKC/NOX pathway -Reduces endothelial proliferation -Modulates nitric oxide signaling
Gut–retina axis dysregulation	gut microbiota, LPS, SCFAs, systemic cytokines	-Dysbiosis increases gut permeability and systemic LPS -Elevating inflammatory mediators that disrupt the BRB and exacerbate DR	-Emerging target in DR management: potential role for microbiome-modulating interventions	-Indirectly modulates gut microbiota and systemic inflammation -Enhances intestinal barrier integrity -Attenuates LPS-driven retinal inflammation (supported by systemic anti-inflammatory effects)

ROS: reactive oxygen species; ETC: electron transport chain; PARP: poly(ADP-ribose) polymerase; GAPDH: glyceraldehyde 3-phosphate dehydrogenase; Nrf2: factor nuclear factor erythroid 2-related factor 2; DR: diabetic retinopathy; ARE: antioxidant response element; HO-1: heme oxygenase-1; AMPK: AMP-activated protein kinase; SIRT1: sirtuin 1; NF-κB: nuclear factor kappa B; IL: interleukin; TNF-α: tumour necrosis factor-alpha; MCP-1: monocyte chemoattractant protein-1; ICAM-1: intercellular adhesion molecule-1; BRB: blood–retinal barrier; NPDR: non-proliferative diabetic retinopathy; PDR: proliferative diabetic retinopathy; NLRP3: NOD-like receptor pyrin 3; RGC: retinal ganglion cell; RNFL: retinal nerve fibre layer; NMDAR: N-methyl-D-aspartate receptor; VEGF: vascular endothelial growth factor; IGF-1: insulin-like growth factor-1; HIF-1α: hypoxia-inducible factor-1 alpha; DME: diabetic macular oedema; PKC: protein kinase C; EndMT: endothelial-to-mesenchymal transition; LPS: lipopolysaccharide; SCFA: short-chain fatty acids.

**Table 2:** Examples of innovative topical formulations with resveratrol in the management of diabetic retinopathy and other ocular diseases.

Formulation Type	Formulation Composition	Physicochemical Characteristics	Indication	Model	Study Type	Advantages	Disadvantages	Author
Polymeric nanoparticles (PEGylated nanoparticles)	RSV, Chitosan—mucoadhesive properties, Sodium tripolyphosphate (TPP)—cross-linker, Polyethene glycol (PEG 2000, 4000, 6000)—surface modifier	Particle size: without PEG: ~14 nm, with PEG: up to ~755 nm (depending on PEG MW and concentration) Zeta potential: Decreased with PEGylation (enhanced biocompatibility) PDI: Increased with PEG Encapsulation efficiency (EE): Up to 91.89%	Glaucoma	Ex vivo rabbit cornea Rabbits	In vitro studies: Drug release: dialysis bag method (12–14 kDa cut-off), PBS pH 7.4 as release medium, magnetic stirring at 600 rpm, temperature 37 °C Initial burst release (~45%), followed by sustained release (up to 12 h), Higher MW PEG = slower release HET-CAM Assay (Ocular Irritation Test) RES-loaded CS NPs: slight irritation after 8 h, RES-loaded PEGylated CS NPs: non-irritant up to 24 h, Conclusion: PEG improves ocular tolerance Ex vivo permeability studies: (excised rabbit cornea) Ex vivo transcorneal permeation, Model: Rabbit cornea with 2–4 mm surrounding sclera, System: Side-by-side Franz diffusion cell Permeability results: PEG-modified CS NPs: 78.34 ± 0.39% permeation, CS NPs: 52.07 ± 1.24%, RES dispersion: significantly less Mechanism: PEGylation enhances transcellular transport, CS increases mucoadhesion and paracellular permeability In vivo studies: Rabbits Ocular Distribution (FITC-labelled study) CS NPs: Accumulate on surface epithelium, PEG-CS NPs: Penetrate deep into cornea and reach retinal choroid PEGylation facilitates deeper tissue penetration	High entrapment efficiency Sustained and controlled drug release Improved corneal permeability and retinal targeting Non-irritant, iso-osmolar, and pH-compatible with the eye Effective IOP reduction over time Reaches the posterior segment (retinal choroid)	Particle size increases with PEG (may affect long-term stability) Entrapment efficiency and drug loading decrease with high PEG concentration The in vivo evaluation was limited to normotensive rabbit models, without validation in a disease-specific glaucoma model	Saravanakumar P et al. (2016) [114]
Polymeric nanogel	RSV, High Molecular Weight Chitosan (300 kDa), extracted from Pleoticus muelleri shrimp waste Sodium, Tripolyphosphate (TPP): Polyanionic cross-linker, Water, Ultrapure grade	Particle Size (DLS):~144 nm PDI: Low, indicating homogeneity Zeta Potential: +32 mV → indicates good colloidal stability Morphology: Spherical, compact structures (TEM) Encapsulation Efficiency (EE): 59 ± 1% Photostability of RSV: Free RSV degraded 29–36% after UV exposure, HCS reduced degradation to ~17%	Non-specific (generally eye diseases)	Retinal Pigment Epithelial cells (RPE) (ARPE-19 cells)	In vitro studies: Model: ARPE-19 human retinal pigment epithelial cells MTT Assay (cytotoxicity): No toxicity up to 1000 µg/mL for any component Cell Morphology: Maintained, as shown by phase-contrast microscopy Inflammatory Response (IL-6 and IL-8 ELISA): No pro-inflammatory response observed (unlike LPS positive control) Cellular Uptake and Colocalisation	Biocompatible, non-toxic, and non-inflammatory to retinal cells High RSV encapsulation efficiency and strong UV protection Suitable size and stability for ocular delivery (~144 nm, +32 mV) Enables intracellular delivery by escaping lysosomal degradation Eco-friendly formulation using natural chitosan from shrimp waste	No in vivo testing performed Limited data on long-term stability and release kinetics Fragile structure under high-force processing (e.g., ultracentrifugation)	Solana Buosi F et al. (2020) [115]
Micellar solution	RSV, Carrier: Soluplus^®^ (Sol), PBS (pH 6.8) Optimal formulation ratio: 18:1 (Sol: RSV by weight)	Size: ~50.1 nm PDI: 0.081 ± 0.002 Zeta Potential: ~−3.5 mV Entrapment Efficiency: ~98.8% pH of final solution: 6.8	Treatment of corneal injuries and other inflammatory diseases of the anterior segment of the eye.	Human corneal epithelial cells (HCECs) Mice Rabbits	In vitro studies: Cytotoxicity: MTT assay on HCECs Cellular uptake: Using Coumarin-6 labelled Sol-Res, observed via fluorescence microscopy and quantified Chemical stability: Degradation under light (photostability); half-life improved from ~117 to ~441 min Storage stability: 12 weeks at 4 °C with >92% Res retention In vivo studies: Ocular tolerance test: Modified Draize test in rabbits with histopathological examination Corneal permeation study: Fluorescence microscopy and HPLC analysis in mouse corneas Corneal wound healing assay: n-heptanol injury model in mice with RT-PCR analysis of cytokines	Improved RSV Stability: Micelle encapsulation significantly increased photostability and chemical stability High Entrapment Efficiency: Nearly complete encapsulation of Res Enhanced Ocular Penetration: Better permeation into corneal epithelium and stroma than free Res Excellent Biocompatibility: Promoted HCEC proliferation, no cytotoxicity observed Strong Wound Healing Effect: Faster epithelial recovery compared to PBS or free Res Anti-inflammatory and Antioxidant Activity: Downregulation of pro-inflammatory cytokines (IL-1β, IL-6, TNFα, COX-2) and upregulation of HO-1, SOD, and SIRT1	Penetration limited to epithelium and stroma: Micelle-associated fluorescence did not reach corneal endothelium Long-term storage stability not fully established: Only short-term data (12 weeks) under refrigerated conditions Formulation colour and scale-up feasibility briefly noted but not deeply evaluated	Li M et al. (2020) [116]
PLGA nanoparticles	RSV, Polymer: PLGA, Acetone (solvent), distilled water, Tween 80 Fluorescent marker: Coumarin-6	Particle size: 102.7 ± 2.8 nm (by DLS); Zeta potential: −47.30 ± 0.9 mV PDI: 0.095 ± 0.003 Entrapment efficiency: 65.21 ± 2.2% Drug loading: 8.3 ± 0.4% Morphology: Spherical, uniform, smooth surface (TEM) Release profile: Sustained release; ~16% in first hour, ~83.4% after 72	Age-Related Macular Degeneration	Retinal Pigment Epithelial cells (RPE) (ARPE-19 cells)	In vitro studies: Model: ARPE-19 human retinal pigment epithelial cells Cytotoxicity: MTT assay at 24 h and 48 h—good biocompatibility (>90% viability for RES-NPs) Cellular uptake: Confocal microscopy using Coumarin-6—confirmed internalization after 2 h Anti-angiogenic activity: VEGF expression measured by ELISA—significant reduction at 24 h and 48 h compared to control	Sustained release allows for prolonged drug availability Reduced VEGF expression, indicating potential anti-angiogenic activity Biocompatibility confirmed by high cell viability Enhanced cellular uptake via endocytosis Minimally invasive potential for intravitreal injection	Study limited to in vitro evaluation No in vivo pharmacokinetic or biodistribution data	Bhatt P et al. (2020) [117]
Mucoadhesive nanoparticles	RSV, Polymers: Lecithin, Chitosan	Particle size: 163.3 nm PDI: 0.254 Zeta potential: +46.4 mV Encapsulation efficiency (EE): 97.03% Cumulative drug release: 96.87% Release kinetics: Zero-order (R^2^ = 0.9897)	Non-specific (generally eye diseases)	The New Zealand albino rabbits (female, weight range of 2.8–3.1 kg)	In vitro studies: Drug release study—Dialysis membrane method under simulated tear fluid conditions Release kinetics—Analysed using zero-order, first-order, and Higuchi models Mucoadhesion evaluation—Turbidimetric assay using mucin dispersion In vivo studies: Ocular pharmacokinetics—Topical administration in rabbits with subsequent measurement of drug levels in aqueous humour using HPLC Pharmacokinetic parameters assessed: AUC_0–6_, MRT Comparison with plain RSV solution to demonstrate improved ocular retention	High entrapment efficiency (97%) ensures maximum drug loading Mucoadhesive properties prolong retention on the ocular surface Positive zeta potential (+46.4 mV) enhances interaction with negatively charged mucins Sustained drug release over time (zero-order kinetics) Improved bioavailability demonstrated by 6.44× increase in AUC_0–6_ and 2.46× increase in MRT compared to RSV solution	Lack of long-term stability studies for the final formulation In vivo testing limited to healthy rabbits—no disease models (e.g., dry eye, inflammation, diabetic retinopathy) were used No evaluation of potential ocular irritation or toxicity beyond pharmacokinetics Scale-up potential and reproducibility in industrial conditions not discussed	Saha M et al. (2021) [119]
Lamellar liquid crystalline gel (gel-based eye drops)	RSV, Matrix components: Glyceryl monooleate (GMO)—lipid phase, Ethanol—solvent (small amount), Water—aqueous phase	Semisolid, transparent or slightly opaque gel Lamellar liquid crystalline gel (confirmed by PLM & SAXS) RSV content: 4.4 mg/g (high drug loading) Crystallinity—Amorphous dispersion (confirmed by PXRD) Viscosity behaviour: Exhibits viscoelastic properties; stable under <100% strain Release profile: Sustained release; ~67% over 7 h Ethanol content ~0.6 g per 1 g gel, structurally embedded with limited contact Mucoadhesiveness: High, enabling prolonged corneal retention (≥90 min) Size (microstructure): Lamellar bilayers; not nanoparticulate—visible gel phase	Corneal neovascularisation (CNV)	Human corneal epithelial cells (HCECs) Ex vivo rabbit cornea male Sprague-Dawley rats	In vitro studies: Drug release study—in saline solution, analysed by HPLC (0–7 h) Cytotoxicity (CCK-8 assay)—on HCEC cells at various concentrations Ex vivo studies: Franz diffusion cell with excised rabbit corneas, Comparison of permeation: ROLGs vs. RHSs In vivo studies (in rats): OCT (Optical Coherence Tomography)—monitoring gel retention on the ocular surface, Sodium fluorescein test—assessment of corneal epithelial integrity Histological analysis (H&E staining)—corneal structure after treatment CNV model (Corneal Neovascularisation, alkaline burn injury)—evaluation of therapeutic effect IHC (Immunohistochemistry)—analysis of VEGF expression in corneal tissue Neovascular area measurement—ImageJ software and slit-lamp imaging	High drug loading capacity Stable lamellar gel structure Enhanced corneal penetration (3× more than suspension) Non-invasive, once-daily administration Safe for the corneal epithelium Significant anti-VEGF and anti-CNV activity Promotes corneal healing and reduces inflammation	Study performed only in rats Long-term stability under ambient conditions was not discussed Ethanol—required for RSV solubilization and structurally embedded in lamellar gel (limited direct contact) Residual ethanol presents a potential risk with prolonged use, so further safety evaluation is recommended for long-term application	Minshu Li et al. (2021) [120]
Cyclodextrin-based nanoparticles	Quercetin (QUE)/RSV; Cyclodextrins (CDs): β-CD, HPβ-CD, RAMEB; Hyaluronic acid (HA) Formulation types: -Binary complexes (QUE/CD and RSV/CD) -Ternary complexes (QUE/CD/HA and RSV/CD/HA)	Particle size RSV/CD/HA: ~82 nm QUE/CD/HA: ~103 nm Spherical nanoaggregates	Dry eye disease	Human Corneal Epithelial Cells (HCECs) Conjunctival epithelial cell (IM-ConjEpi) lines	In vitro studies: Assessment of cytoskeletal morphology and cell viability In vitro ROS assay: Evaluation of antioxidant activity (DCF-DA method)	Significantly improved solubility and chemical stability of RSV and QUE in complexes Formation of stable nanoaggregates suitable for ophthalmic delivery Strong antioxidant effect (>95% intracellular ROS scavenging) in both cell lines No cytotoxic effects observed on HCE and IM-ConjEpi cells Promising potential for topical treatment of Dry Eye Disease (DED)	In vitro study only—no in vivo data on efficacy or ocular penetration Short-term stability assessment—long-term characterization is needed Further validation required through clinical studies or dry eye models	Krstić L et al. (2022) [121].
Micellar solution	RSV, Carriers: Pluronic^®^ F127 (Poloxamer 407)—amphiphilic copolymer Casein (0.1%)—milk protein, Phosphate buffer (pH 7), ethanol, water, propylene glycol Formulations: Single micelles: Pluronic^®^ F127 (5–15 mM) Mixed micelles: Pluronic^®^ F127 + casein	Physicochemical Properties Particle size: 2.4–32.7 nm Zeta potential: Pluronic^®^ micelles: near 0 mV Mixed micelles: –0.1 to –2.2 mV (depending on composition) Formulation pH: 7.0–7.3 RSV solubility: Increased up to 50–57 times (up to 9.36 mg/mL) Stability: Stable for 30 days at 4 °C Rheology: Sol-to-gel transition at 23.9–27.1 °C Mucoadhesion: Significantly increased on the corneal surface	Non-specific (generally eye diseases)	Ex vivo porcine eye globes Rabbits	In vitro/in vivo safety HET-CAM assay: No irritation observed in any formulation Zebrafish FET test: 5 and 10 mM Pluronic^®^: safe, 15 mM and mixed micelles: mildly toxic Antibacterial activity: Active against S. aureus and P. aeruginosa, stronger effect with higher RSV concentrations, Pluronic^®^ alone showed a mild antibacterial effect Ex vivo permeability (porcine cornea and sclera) Higher accumulation in the sclera than in the cornea, Mixed micelles: slower onset and lower permeability Single micelles (P10R): better permeation and tissue uptake In vivo studies in rabbits: single micelles—good ocular tolerance (no irritation) Delivery of RSV to both anterior and posterior eye segments, detectable RSV in tears, cornea, sclera, and retina even 8 h post-instillation	High RSV solubilization and stability Physiological pH and non-irritant Easy administration (liquid-to-gel transition) Effective tissue penetration and prolonged retention Antioxidant and antibacterial activity Potential alternative to preservatives in eye drops	Casein-containing mixed micelles showed increased toxicity in zebrafish embryos Lower scleral permeability with mixed micelles Further long-term safety evaluation needed for mixed systems	Vivero-Lopez M et al. (2022) [122]
Chitosan-coated liposomes	RSV, Core lipid carrier: Flexible liposomes prepared using egg yolk phospholipid (EYPC), cholesterol, and sodium cholate Surface coating: TMC, Stabiliser: Sodium cholate for liposome flexibility	Size: ~92.13 ± 0.70 nm (before TMC coating) PDI: 0.223 ± 0.026 Zeta potential: Positive after TMC coating Shape: Uniform spherical shape confirmed by TEM	Blue-light-induced retinal damage	Retinal Pigment Epithelial cells (RPE) (ARPE-19 cells) Mices	In vitro studies: Cell model: ARPE-19 human retinal epithelial cells Cell viability under oxidative stress (H_2_O_2_ challenge) Mitochondrial membrane potential protection (JC-1 assay) Uptake and distribution studies In vivo studies: Animal model: Mice subjected to blue-light-induced retinal damage (Retinal targeting via fluorescent tagging), Histopathology (frozen retinal sections), safety and efficacy through tissue observation and damage attenuation)	Improved ocular penetration: Positively charged TMC enhanced adhesion to ocular tissues and deeper retinal penetration. Effective delivery to the fundus: Confirmed RSV delivery to the posterior segment. Protection against oxidative stress: Reduced blue-light-induced mitochondrial damage and retinal cell injury. Stability: Formulations remained stable in artificial tear fluid.	Lack of long-term in vivo data: Chronic toxicity or prolonged efficacy was not addressed. Scalability and formulation complexity: Use of TMC and flexible liposomes may pose challenges in mass production. Exact RSV release kinetics and in vivo bioavailability were not deeply explored	Gu H et al. (2023) [123]
In-Situ Thermoresponsive Hydrogel	RSV, Carrier polymer: PLGA-PEI, Stabiliser: Polysorbate 80 Gel matrix: Poloxamer 407 (20% *w*/*v*), thermoresponsive hydrogel	particle size: 189.0 ± 3.2 nm Zeta potential: +21.5 ± 1.8 mV Entrapment efficiency (EE): 83.6 ± 1.7% Gelation temperature: ~32 °C (in situ gelation) Release profile: 68% of RSV released within 24 h (sustained release) Rheology: Increased viscosity after gelation (solid-like behaviour)	Dry eye disease	Human corneal epithelial cells (HCECs) Ex vivo porcine eye globes	In vitro studies: Model: HCECs under hyperosmotic stress Tests performed: Cell viability (MTT assay) Apoptosis detection (DAPI staining, immunofluorescence for BAX/BCL-2) Oxidative stress analysis: ROS and lipid peroxidation (MDA) Gene expression (qRT-PCR): IL-1β, IL-6, TNF-α, SIRT1 Western blotting: SIRT1 and NF-κB protein expression Cellular uptake of fluorescently labelled nanoparticles Ex vivo studies: Porcine eye—confirmed penetration and retention on the corneal surface via confocal microscopy	In situ gelation provides prolonged residence time on the ocular surface Cationic nanoparticles enhance mucoadhesion and epithelial penetration Strong antioxidant and anti-inflammatory activity—reduced ROS, MDA, and pro-inflammatory cytokines Biocompatible—no cytotoxic effects observed in HCECs Potential for reduced dosing frequency in Dry Eye Disease treatment	Lack of in vivo pharmacodynamic and pharmacokinetic data Long-term stability of the hydrogel under storage conditions not evaluated Possible cytotoxicity of PEI at higher concentrations (not observed in this study) Further validation required in animal models and clinical settings	De Luca I et al. (2023) [124]
Microneedles	RSV, Nanoparticle core: PLGA, Targeting shell: RPE cell membrane, Microneedle matrix: Optional excipients/dyes (for tracking): Rhodamine B, Coumarin-6	Uncoated RSV-loaded nanoparticles: 156.63 ± 1.95 nm RPE-coated nanoparticles (RmNP-RSV): 169.23 ± 1.43 nm The ~15 nm increase reflects the successful coating with RPE membrane vesicles.	Age-related macular degeneration	Retinal Pigment Epithelial cells (RPE) (ARPE-19 cells) Rabbits	In vitro studies: Cell uptake studies confirmed enhanced internalisation of RmNP-RSV in RPE cells. Antioxidant and anti-inflammatory assays showed reduction of ROS and pro-inflammatory cytokines such as IL-6 and IL-8 in ARPE-19 cells. In vivo studies: The rabbit model of NaIO3-induced dry AMD was used. Administration of MN/RmNP-Res showed significant preservation of retinal architecture, reduced oxidative stress and inflammation, and reduced retinal degeneration. Imaging (OCT, fundus photography) and histological analysis (H&E staining) confirmed therapeutic benefit.	Targeted delivery to retinal tissue via homologous RPE membrane coating Minimally invasive, painless, and avoids complications of intravitreal injections Efficient penetration of the blood–retinal barrier Enhanced RSV bioavailability and tissue retention Improved safety profile and reduced systemic exposure	The study was limited to animal models; clinical translation requires further validation. Manufacturing complexity due to the need for RPE membrane extraction and microneedle fabrication The stability of biomimetic coatings under storage conditions was not addressed	Liu Y et al. (2023) [128]
Nanosuspension	CMC, RSV, Tween 20, Span 20, ethanol DMSO, glycerin	Particle size 304 ± 81 nm PDI: 0.225 ± 0.036 Morphology: Spherical	Diabetic Retinopathy	Human microvascular retinal endothelial cells (HMRECs)	In vitro studies: Cytotoxicity—MTT assay Proliferation -Cell proliferation assay Migration—Scratch wound healing assay	No cytotoxic effect at concentration < 18.75 µM Significantly reduces cell proliferation and migration at 37.5 µM → clinically relevant for inhibiting neovascularisation in diabetic retinopathy Physically stable nanosuspension with homogeneous particle size Topical administration—non-invasive alternative to injections	In vitro study only—results based solely on cell models No in vivo testing—actual retinal penetration remains unknown Short-term evaluation—lacks data on long-term stability or pharmacokinetics	Gonzales-Perez J et al. (2024) [126]
SNEDDS	RSV/Melatonin (MLT); SNEDDS excipients: Capryol 90 (Oil phase), Kolliphor RH 40 (Surfactant), Transcutol HP (Co-surfactant:)	Droplet size: 42–85 nm PDI <0.2 pH, viscosity, dilution stability Emulsification time: ~40 s pH/osmolarity: Suitable for ocular use (pH 6.9–7.5; 0.281–0.320 Osm/kg)	Non-specific (posterior eye diseases)	Rabbit corneal epithelial cell line (SIRC)	In vitro studies: Solubility and dissolution of RSV and MLT tested under physiological pH Cytocompatibility: SIRC Antioxidant activity evaluated under oxidative stress conditions	Self-nanoemulsifying—forms a stable nanoemulsion upon contact with the tear fluid Ultra-small droplets (<50 nm): allow deep ocular penetration, potentially even beyond anterior segment Rapid emulsification: ~40 s ensures quick action before tear clearance High loading efficiency for both RSV and MEL (>90%) Ocular-friendly properties: clear, non-irritating pH/osmolarity and maintained viscosity Promising safety profile: cytocompatible with corneal cells	In vitro only: no in vivo or clinical efficacy data yet Unknown posterior distribution: actual delivery to retina/vitreous not tested Short-term stability tests only: longer-term performance in real-world conditions remains unassessed Mucoadhesion minimal: slight interaction with mucin noted, but may need enhancement for prolonged retention	Zingale E et al. (2024) [125]
NLC	RSV, Solid lipid: Glyceryl monostearate, Liquid lipid: Soybean oil, Surfactants: Tween 80, Poloxamer 407 Aqueous phase: Purified water	Particle size (DLS): 104.47 ± 13.32 nm PDI = 0.394 ± 0.070 Zeta potential (ZP): –1.63 ± 0.29 mV Entrapment efficiency (EE): 85.88 ± 0.32%	Non-specific (generally eye diseases)	Goat cornea	In vitro studies: Release testing: Franz diffusion cell using simulated tear fluid/methanol (1:1), maintained at 38 °C. Release profile: Initial burst release followed by sustained release over 24 h Ex vivo studies: Model: Isolated goat cornea in Franz diffusion cell setup—Improvement over suspension: Over 11-fold enhancement in transcorneal permeation	Nanometric size enables effective corneal penetration High drug entrapment efficiency Sustained release behaviour extends drug residence time Significantly enhanced bioavailability and corneal absorption Potential for reduced dosing frequency	No in vivo testing conducted Mildly negative zeta potential may affect long-term colloidal stability UV spectroscopy has limited sensitivity compared to chromatographic methods	Chakole CM et al. (2024) [118]
Protein-based polymeric drug delivery systems	Active compounds: Quercetin (QUE), RSV, or their combination Polymeric carrier: Elastin-like polymers (ELPs)	Dry form: Microparticles In physiological conditions (37 °C): Nanoparticles with an average diameter of QUE-loaded: 56.7 ± 1.0 nm RSV-loaded: 61.5 ± 2.6 nm	Dry eye disease	Human Corneal Epithelial Cells (HCECs) Ex vivo porcine eye globes	In vitro studies: Biocompatibility HCECs Intracellular antioxidant activity (ROS scavenging assays) Cellular uptake tracking using dual fluorescent labelling Ex vivo: corneal targeting in porcine eye globes (time-dependent delivery to corneal epithelium)	Eco-friendly production method (no organic solvents) Stimuli-responsive transformation enabling improved bioavailability Sustained release of polyphenols Excellent biocompatibility and enhanced intracellular delivery Efficient, targeted delivery to the corneal epithelium	No in vivo studies reported Pharmacokinetic parameters and long-term stability data are not provided	Krstić L et al. (2025) [127]

SNEDDS—Self-Nanoemulsifying Drug Delivery System—is a system that spontaneously forms nanoemulsions upon contact with aqueous media; NLC—nanostructured lipid carriers; AUC_0–6_ (area under the curve); RSV—resveratrol; MRT (mean residence time);PLGA-PEI (acetylated poly (D,L-lactic-co-glycolic acid)–polyethyleneimine), RPE—Retinal pigment epithelial cell membrane (for biomimetic coating); TMC—Trimethylated chitosan is a positively charged mucoadhesive polymer, used to coat the flexible liposomes through electrostatic adsorption; HET-CAM Assay (Hen’s Egg Test—Chorioallantoic Membrane) to evaluate the ocular irritancy potential of formulations without using live animals; CS—chitosan, PEG—Polyethylene glycol.

**Table 3:** Comparison of Current Therapies for Diabetic Retinopathy and the Potential Role of Resveratrol.

Therapy	Mechanism of Action	Advantages	Limitations	Complementary or Enhancing the Role of Resveratrol
Anti-VEGF Agents (ranibizumab, aflibercept, bevacizumab)	-Neutralise VEGF to inhibit neovascularisation and reduce vascular permeability	-Clinically validated -Effective in PDR and DME -Reduces macular oedema and vision loss	-Requires repeated intravitreal injections -Limited penetration in early-stage DR -Does not address neuroinflammation or oxidative stress	-Suppresses VEGF and HIF-1α via multi-pathway modulation (Nrf2, AMPK, SIRT1) -Acts on the upstream angiogenic triggers -Potential for oral or topical use -Longer-term neurovascular protection without invasive procedures
Laser Photocoagulation	-Ablation of ischemic retinal tissue to reduce VEGF production and prevent neovascularisation	-Proven efficacy in PDR -Reduces the risk of severe vision loss	-Causes permanent retinal damage; -Loss of peripheral and night vision -Not effective for DME or neurodegeneration	-Offers non-destructive, cytoprotective, and neurovascular preservation -Inhibits VEGF without damaging the viable retinal tissue
Intravitreal Corticosteroids (triamcinolone acetonide, dexamethasone implants)	-Suppresses inflammatory cytokines and stabilises the blood–retinal barrier	-Effective in reducing DME and retinal inflammation -Sustained-release options available	-Associated with ocular hypertension -Cataract formation -Repeated injections increase the risk	-Inhibits NF-κB and NLRP3 inflammasome pathways -Provides anti-inflammatory protection with a lower risk of ocular side effects
Surgical Intervention—vitrectomy	-Removes vitreous haemorrhage or tractional membranes to restore retinal architecture	-Effective in resolving complications of advanced DR (TRD, VH)	-Highly invasive -Does not prevent progression or target early mechanisms	-Targets upstream pathophysiological pathways to potentially delay or prevent surgical indications
Emerging Systemic Therapies (RAS inhibitors, fenofibrate, anti-inflammatory agents)	-Modulate systemic risk factors: blood pressure, dyslipidaemia, and systemic inflammation	-Reducing the progression of DR -Oral administration -Systemic disease control	-Limited direct retinal bioavailability -Insufficient neuroprotection	-Exerts systemic and retinal effects through AMPK, SIRT1, and anti-inflammatory pathways -Synergistic with systemic agents
Resveratrol	-Multifunctional: antioxidant (Nrf2/HO-1), anti-inflammatory (NF-κB, NLRP3), anti-angiogenic (VEGF/HIF-1α), neuroprotective (SIRT1, AMPK)	-Addresses multiple early DR mechanisms -Neurovascular protection -Non-invasive potential -Multi-targeted effects	-Low aqueous solubility and systemic bioavailability -Dose standardisation needed -Limited clinical validation	-Promising adjunct or preventive agent -Emerging nanoformulations and implants aim to overcome pharmacokinetic challenges

VEGF: vascular endothelial growth factor; PDR: proliferative diabetic retinopathy; DR: diabetic retinopathy; DME: diabetic macular oedema; HIF-1α: hypoxia-inducible factor-1 alpha; Nrf2: factor nuclear factor erythroid 2-related factor 2; AMPK: AMP-activated protein kinase; SIRT1: sirtuin 1; NF-κB: nuclear factor kappa B; NLRP3: NOD-like receptor pyrin 3; TRD: tractional retinal detachment; VH: vitreous haemorrhage; RAS: renin-angiotensin system.

**Table 4:** Resveratrol in Diabetic Retinopathy: Pathophysiological Targets, Molecular Pathways, and Translational Potential.

Pathophysiological Aspect	Scientific Basis	Mechanism of Action	Proposed Clinical Strategy	Anticipated Therapeutic Benefit
Vascular Dysfunction and BRB Breakdown	-Hyperglycaemia- induced VEGF overexpression and oxidative stress led to BRB disruption, vascular leakage, and neovascularisation.	Downregulates VEGF and ICAM-1 -Promotes pericyte survival -Stabilises endothelial junctions	-Adjunct low-dose anti-VEGF therapy -OCTA and serum VEGF monitoring.	-Reduced injection burden -Enhanced vascular integrity -Delayed progression to PDR
Neurodegeneration and RGC Loss	-Early DR involves retinal ganglion cell apoptosis, synaptic loss, and neuroinflammation	-Activates SIRT1 and AMPK -Enhances autophagy -Suppresses microglial activation	Combined use with neuroprotectives (citicoline, brimonidine) -Biomarker-driven personalisation	-Improved visual function and RGC preservation in early DR stages
Gut–Retina Axis Dysregulation	-Diabetes-associated dysbiosis increases systemic inflammation and metabolic endotoxemia, worsening retinal damage	-Modulates gut microbiota -Reduces LPS and systemic cytokines -Activates Nrf2	-Co-administration with prebiotics/ probiotics -Microbiome-based patient stratification	-Reduced systemic inflammation -Improved metabolic control and DR outcomes
Limited Bioavailability and Retinal Penetration	-Rapid metabolism and low solubility limit the systemic and ocular efficacy of resveratrol	-Nanoencapsulation, intravitreal -Sustained-release systems -Prodrug strategies	-Development of PEGylated nanoparticles -Ocular pharmacokinetics and biodistribution studies	-Increased intraocular drug levels -Prolonged therapeutic effect -Reduced dosing frequency
Lack of Standardisation in Clinical Translation	-Variability in dose, endpoints, and trial design impedes clinical applicability	Targets multiple pathways -Potential synergy with standard DR treatments	-Standardised protocols -Biomarker-based stratification -Multicentre RCTs.	Enhanced evidence base -Personalised medicine integration -Adopting the guidelines

VEGF: vascular endothelial growth factor; ICAM-1: intercellular adhesion molecule-1; OCTA: optical coherent tomography angiography; BRB: blood–retinal barrier; PDR: proliferative diabetic retinopathy; DR: diabetic retinopathy; SIRT1: sirtuin 1; AMPK: AMP-activated protein kinase; RGC: retinal ganglion cell; LPS: lipopolysaccharide; Nrf2: factor nuclear factor erythroid 2-related factor 2; RCT: randomised clinical trials.
